# Bicycle Touring 480 km in Seven Days: Effects on Body Composition and Physical Fitness—A Case Study

**DOI:** 10.3390/ijerph19052550

**Published:** 2022-02-23

**Authors:** David Zúñiga-Moreno, Francisco J. Amaro-Gahete, Palma Chillón

**Affiliations:** 1 PROFITH “PROmoting FITness and Health through Physical Activity” Research Group, Department of Physical Education and Sport, Faculty of Sport Sciences, University of Granada, 18071 Granada, Spain; david61093@gmail.com (D.Z.-M.); pchillón@ugr.es (P.C.); 2 EFFECTS-262 Research Group, Department of Physiology, Faculty of Medicine, University of Granada, 18016 Granada, Spain

**Keywords:** bicycle touring, body composition, fat mass, lean mass, physical fitness, cardiorespiratory fitness

## Abstract

Bicycle touring as a modality of physical activity that involves whole-body cardiorespiratory and metabolic functions could be a potential strategy to improve body composition and cardiorespiratory fitness. Therefore, the aim of the current study was to investigate the effects of 7-days bicycle touring activity on body composition and physical fitness. A total of 13 individuals (three women) participated in this quasi-experimental study. The participants were evaluated at baseline and were tracked for up to 12 days after the intervention. The intervention consisted of a 480 km bicycle touring route performed over 8 days. Body weight and body composition (i.e., fat mass and lean mass) were assessed using a bioelectrical impedance analyser. Physical fitness was measured using the International Fitness Scale questionnaire. We conducted a repeated-measures analysis of variance to determine changes in body weight and body composition and paired sample *t*-tests to analyse changes in physical fitness. Significant differences in fat mass were observed between the baseline and both post-intervention (*p* = 0.003) and re-test values (*p* = 0.031). Significant differences were also noted in lean mass between the baseline and post-intervention values (*p* = 0.003), whereas no significant changes were observed when compared the baseline and re-test values (*p* = 0.178). Significant differences were obtained in cardiorespiratory fitness when comparing the baseline with the post-intervention values (*p* = 0.016), whereas no significant differences were noted in general fitness, muscular strength, speed/agility, and flexibility (all *p* > 0.05). In conclusion, a 7-day bicycle touring intervention can significantly reduce fat mass and increase lean mass and cardiorespiratory fitness in healthy individuals.

## 1. Introduction

There is widespread scientific evidence supporting the health-related benefits of physical activity (PA), being a common message of public health within health promotion settings [1,2,3,4]. Indeed, high physical activity levels have been associated with a marked reduction of (i) cardiometabolic disease incidence and (ii) risk for premature mortality [5,6,7,8]. In healthy individuals, PA has also been recommended not only for the maintenance or even the decrease of weight and fat mass, but also for fitness [9,10,11,12]. In this sense, routine PA behaviours have been associated with a marked decrease of premature risk of mortality and a reduced incidence of more than 26 chronic medical conditions (e.g., psychiatric diseases, neurological diseases, metabolic diseases, cardiovascular diseases, pulmonary diseases, musculo-skeletal disorders and cancer) [3,13,14].

Bicycle touring is defined as an activity executed by a person or group of people that leave their home town or country for a period not less than 24 h or one night, usually for a vacation or holiday, and using a bicycle as a mode of transport [15]. In addition, it may include the use of transport support services and formal and/or informal accommodation [15,16]. There are six general characteristic of bicycle touring: (1) being away from home, (2) a duration from one night to multiple days, (3) being non-competitive, (4) considering cycling as its main purpose, (5) being performed in an active context, and (6) being understood as a recreation/leisure activity [15,17]. Bicycle touring is a sustainable form of transport and may offer important benefits for both individual tourists’ health and the environment [18,19]. In this sense, it has been considered as an alternative solution to two major problems for humanity—especially in developed countries: climate change (i.e., motor vehicles are a major source of environmental and noise pollution in urban areas accounting for >70% of environmental pollution and >40% of greenhouse gases in European cities) and the obesity pandemic [20,21]. In addition, using cycling as a mode of transportation contributes to achieving 14 of the 17 sustainable development goals from Agenda 2030 [22].

Physical fitness, specifically cardiorespiratory fitness (CRF), is a powerful predictor of all-cause mortality, cardiovascular disease, and cancer [4,23,24], and is defined as a set of attributes that people have or achieve related to the ability to perform PA. CRF is a health-related component of physical fitness associated with the ability of circulatory and respiratory systems to supply fuel during sustained PA and to eliminate fatigue products after supplying fuel [25]. Increasing PA levels is a well-known strategy to improve physical fitness in the majority of individuals [14]. In this context. bicycle touring implies an important load on the human body requiring initial training, practice, good health and fitness, and should be recommended to relatively fit individuals for preventing obesity and other chronic medical conditions in the long-term [15]. Indeed, it has been demonstrated that this kind of intervention is appropriate to enhance cardiorespiratory fitness not only in normal-weight individuals but also in overweight adults [26].

Cycling is a modality of PA that involves whole-body cardiorespiratory and metabolic functions over a wide range of intensities, leading itself to many potential health benefits [18,26,27]. The majority of these studies have been conducted in a framework of active commuting, showing an association between this activity and a reduction in cardiovascular risk [28], all-cause mortality risk [29], and a decrease in body weight [30]. Even e-bikes have been suggested as an effective tool to improve CRF similar to that obtained by conventional bicycles, becoming a potential solution for individuals who are not able to easily perform PA [31,32]. Bicycle touring, as a type of PA, could be a potential strategy to improve body composition and CRF. To the best of our knowledge, there is no previous evidence in the scientific literature regarding the health-related effects of bicycle touring interventions. Vujko et al. [33] reported an increment of self-perceived health status and quality of life in response to bicycle touring activities. However, they did not analyse the effects of bicycle touring on physical health parameters.

According to the above-mentioned background, it seems clear that bicycle touring may induce an improvement in both body composition and physical fitness through an increase in PA levels. However, since the majority of studies have been carried out on individuals that performed the cycling as commuting, it is unknown whether these effects occur after the application of a bicycle touring intervention performed at low intensity, over a prolonged period of time, and extended to a few days’ duration. Therefore, the objective of the current study was to investigate the effects of a 7-day bicycle touring activity on body composition and physical fitness in healthy individuals.

## 2. Materials and Methods

### 2.1. Participants

The participants of the present study were a total of 13 individuals (3 women), aged between 21 to 58 years old, where 10 of them were new to participation in bicycle touring routes. All of the participants were previously selected for the project “BiciConecta UGR” (biciconectaugr.com (accessed on 1 January 2022)); it was a convenience and variety sample including 7 lecturers and research staff, 5 students, and 1 entrepreneur from the University of Granada. The inclusion criteria were: (i) to be a member of the University of Granada, (ii) to present with no acute and/or chronic diseases, (iii) to show a health status that was medically confirmed to be suitable to completing the present intervention, and (iv) to self-report having previous cycling experience as a recreational activity (i.e., cycling as a mode of transportation and cycling >1 time per week during at least the previous 6 months).

### 2.2. Study Design

This was a non-randomized uncontrolled single arm study. The participants were evaluated for anthropometry and body composition at three different times and for physical fitness at two different times. The study was performed according to the latest revised guidelines of the Declaration of Helsinki, and was approved by the Human Research Ethics Committee of the University of Granada (1542/CEIH/2020). All participants were instructed to maintain their usual nutritional and PA habits.

### 2.3. Procedures

Before the beginning of the current intervention, the potential participants received oral and written information about the study’ procedures, ensuring the confidentiality of the collected data and their exclusive use for research purposes. All participants decided to voluntarily participate and they filled out and signed an informed consent form. The research staff accompanied the participants during the whole bicycle touring intervention, efficiently solving any problems derived from the measurement devices. At the end of the study, an individual report was sent to each participant, following ethical guidelines.

### 2.4. Intervention

The bicycle touring intervention was carried out from the 1 to the 8 October 2019, starting in Granada and ending in Ceuta.

**Figure 1:**
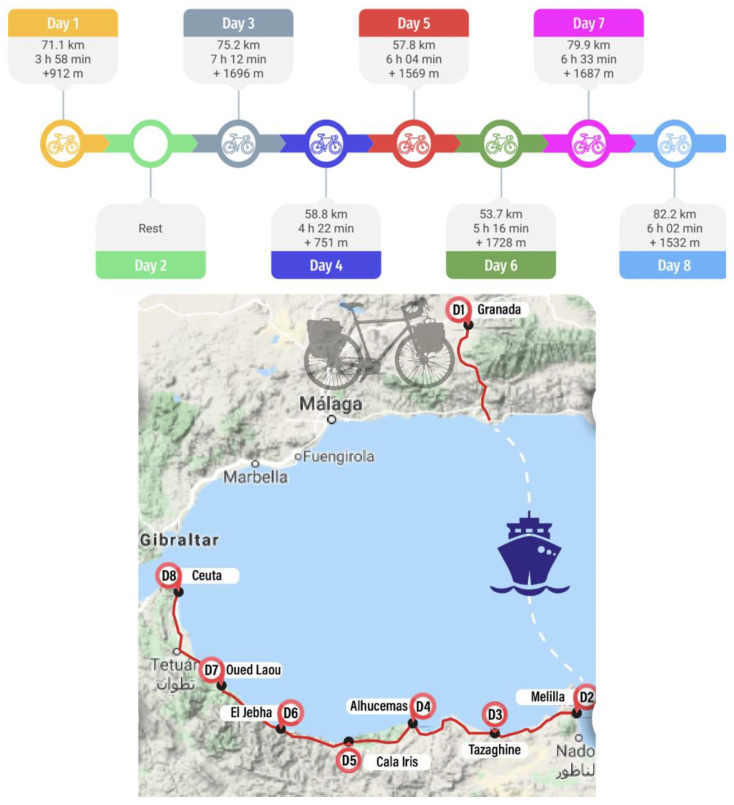
Description of the route.

A 480 km total bicycle touring route was performed over 8 consecutive days (i.e., 7 days of cycling and 1 day resting). Every day had different distances and inclinations, and the route crossed mostly coastal areas around the Mediterranean Sea. A description of the route, including the exact distance and inclination per day, and the geographical location of the route can be found in Figure 1. The route combined smooth and mountainous roads, using asphalt, sandy, and grass paths. Average temperature and humidity were 19.7 °C and 62%, respectively. Neither wind nor rain were noted during the intervention. Moreover, the entire route was performed cycling in a group and within a variable rhythm attending to uncertain situations (i.e., mechanical problems, personal needs such as being hungry or needing rest, mistakes in the route, voluntary stops, etc.). Each participant had their own personal bikes; they were all MTB bikes and no one was carrying any extra weight, as the support van carried everything they needed. After completing the above-mentioned bicycle touring route, the participants were asked to rest, avoiding any kind of physical activity after completing the post-intervention assessment.

### 2.5. Outcomes

#### 2.5.1. Anthropometry and Body Composition

Body weight and body composition (i.e., fat mass and lean mass) were assessed using a bioelectrical impedance analyser (Inbody R20, Biospace, Seoul, Korea), strictly following the manufacturer’ guidelines. The participants were evaluated after emptying their bladder, barefoot with light clothes, and free of metal-based accessories. The validity and reliability of this instrument has been previously established, obtaining <3% of error when fat mass (%) is assessed by bioelectrical impedance vs. dual X-ray absorptiometry [34,35]. Body height was measured by asking the participants how tall they were the last time they were measured. After that, BMI was calculated as body weight (kg) divided by body height^2^ (m^2^).

Body weight and body composition were measured at 3 different times (i.e., at the baseline, the day after finishing the intervention, and 7 days after the end of the intervention). Body height was measured at baseline and the value remained constant in the 3 measurement times.

#### 2.5.2. Physical Fitness

Physical fitness was measured using the self-report questionnaire “International FItness Scale” (IFIS). This questionnaire was developed in Spain by the HELENA group and validated in Spanish adults [24,36]. It is structured in five different dimensions related to physical fitness components: (i) general fitness, (ii) CRF, (iii) muscular strength, (iv) speed/agility, and (v) flexibility. Partial and total scores were obtained by Likert scales considering the following answers: (i) “Very poor”, (ii) “Poor”, (iii) “Medium”, (iv) “Good”, or (v) “Very good”.

Physical fitness was determined twice (i.e., at the baseline and 12 days after the last day of the intervention) using a Google Forms questionnaire (Alphabet Co., Mountain View, CA, USA).

#### 2.5.3. Heart Rate

Heart rate was continuously monitored during the whole intervention using a Polar RS300x (Polar Electro Oy, Kempele, Finland) or a Polar M430 device (Polar Electro Oy, Kempele, Finland). Mean heart rate of each intervention day was considered for the final analysis.

The researcher staff carried both extra monitors and chest straps in case there were any problems during the recording. On the first day of the activity, participants were taught to start, pause, and end the activity with their own devices. Additionally, the researcher checked the proper functioning of each device at the beginning, at any stop, and at the end of the activity.

#### 2.5.4. Perceived Exertion

PA intensity was controlled using the Borg scale 6–20 [37]. The Borg scale ranged from 6 to 20, where “6” means no exertion and “20” maximal exertion. The Rate of Perceived exertion (RPE) was evaluated at the end of each daily activity using a specific image. Participants individually gave their scores and they were recorded with a mobile phone.

#### 2.5.5. Emotion (Affective Slider)

Self-reported emotion was additionally controlled by a Likert scale aiming to assess pleasure and arousal. These scales range from −5 to +5, where −5 rates as unhappy and +5 as happy for pleasure, and as sleepy and wide-awake for arousal, respectively [38]. Emotion was evaluated at the end of each daily activity using a specific image. Participants individually gave their scores and were recorded with a mobile phone.

### 2.6. Statistical Analysis

Sample size calculations were calculated by G*Power software (Heinrich-Heine-Universität Düsseldorf, Düsseldorf, Germany), based on a minimum predicted 1% change in fat mass between pre- and post-intervention values, and with an expected standard deviation of 0.5%. A sample size of 11 participants was predicted to provide a statistical power of 80%, considering a type I error of 0.05. We recruited 13 participants per group, accounting for a potential loss of 25% at follow-up.

Descriptive parameters are expressed as mean ± standard deviation unless otherwise stated. We checked the normality of our data by the Shapiro–Wilk test, visual check of histograms, and Q-Q plots. As no significant interaction was obtained by sex, we fitted all models including men and women together.

Unpaired sample *t*-tests were performed to determine differences in age, anthropometry, body composition, and physical fitness between men and women.

We conducted a repeated-measures analysis of variance to determine changes in body weight, fat mass, and lean mass between baseline, post-intervention, and re-test (i.e., 7 days after the intervention). Paired sample *t*-tests were conducted to analyse changes in physical fitness between baseline and post-intervention.

Pearson’s correlation analysis was used to study whether changes in body composition were dependent of the participants’ BMI status.

We established a level of significance of *p* < 0.05. The Statistical Package for Social Sciences (SPSS, v. 22.0, IBM SPSS Statistics, IBM Corporation) was selected to perform the statistical analysis. GraphPad Prism 5 (GraphPad Software, San Diego, CA, USA) was used for generate graphical plots.

## 3. Results

The descriptive characteristics of the participants, separated by sex, are shown in Table 1. The participants reached an average heart rate of 115 ± 5 ppm during the 5.6 ± 0.5 h of each completed stage.

**Figure 2:**
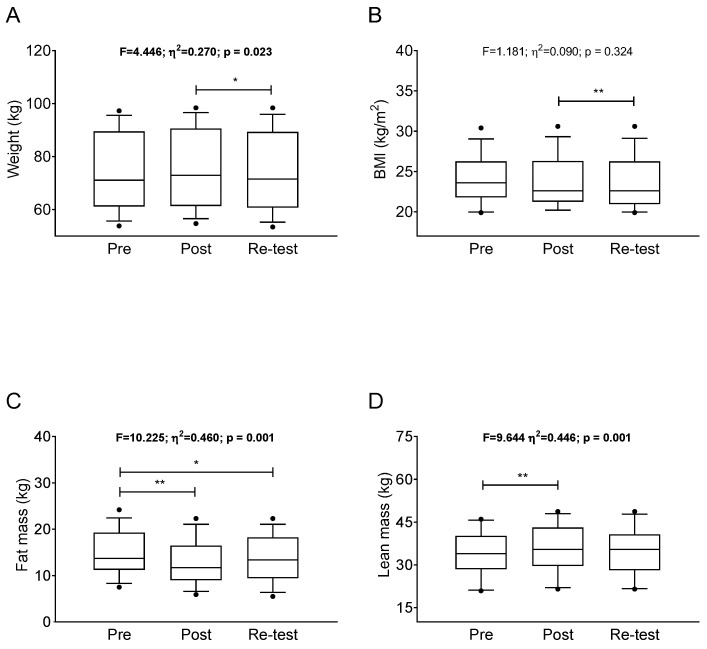
Changes in anthropometry (i.e., weight (**A**) and BMI (**B**)) and body composition (i.e., fat (**C**) and lean mass (**D**)) after the bicycle touring intervention. *p* value derived from repeated-measures analysis of variance. Data are shown as mean ± 95% confident intervals. * *p* < 0.05; ** *p* < 0.01.

Significant differences in body weight were observed between the post-intervention and re-test (Δ = −0.800 ± 0.225; *p* = 0.012; Figure 2A), whereas no significant differences were noted in body weight either between the baseline and post-intervention (Δ = 0.508 ± 0.300; *p* = 0.348; Figure 2A), nor between the baseline and re-test (Δ = −0.292 ± 0.284; *p* = 0.970; Figure 2A). Significant differences in BMI were found between the post-intervention and re-test (Δ = −0.262 ± 0.074; *p* = 0.004; Figure 2B), whereas no significant differences were noted in BMI between either the baseline and post-intervention (Δ = 0.085 ± 0.292; *p* = 0.777; Figure 2B), nor between the baseline and re-test (Δ = −0.346 ± 0.274; *p* = 0.230; Figure 2B). Significant differences were observed in fat mass between the baseline and post-intervention (Δ = −2.092 ± 0.479; *p* = 0.003; Figure 2C), and between the baseline and re-test (Δ = −1.492 ± 0.492; *p* = 0.031; Figure 2C), whereas no significant differences were noted in fat mass between post-intervention and the re-test (Δ = 0.600 ± 0.458; *p* = 0.645; Figure 2C). Significant differences were also found in lean mass between the baseline and post intervention (Δ = 1.638 ± 0.383; *p* = 0.003; Figure 2D), whereas no significant differences were observed in lean mass between either the baseline and re-test (Δ = 0.746 ± 0.358; *p* = 0.178; Figure 2D), nor between the post-intervention and re-test (Δ = −0.892 ± 0.379; *p* = 0.109; Figure 2D).

**Figure 3:**
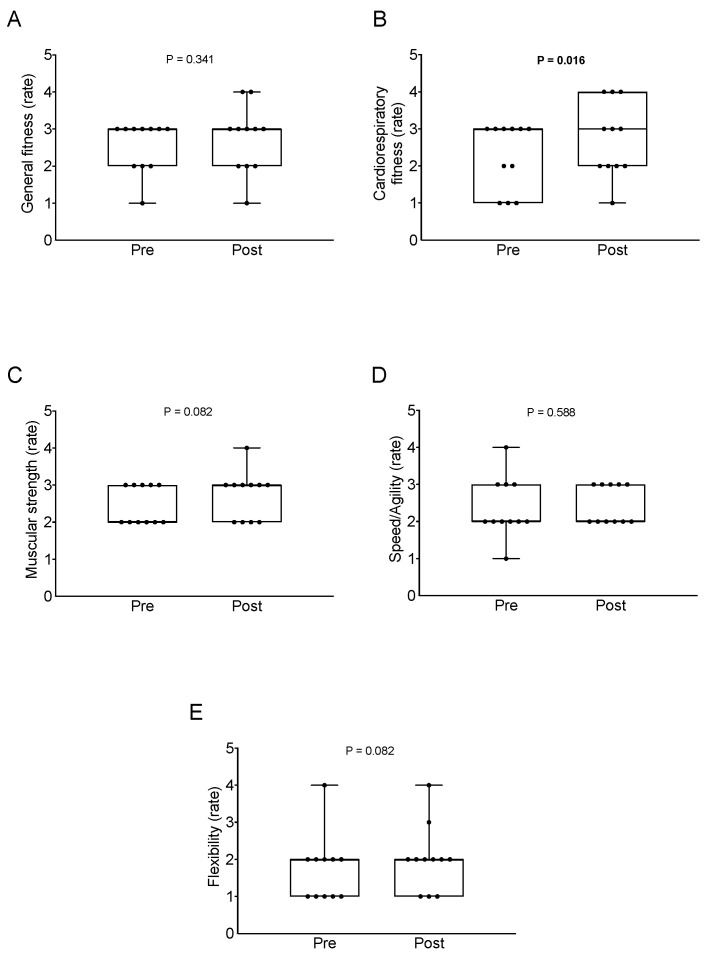
Changes in physical fitness (i.e., general fitness (**A**), cardiorespiratory fitness (**B**), muscle strength (**C**), speed/agility (**D**), and flexibility (**E**)) after the bicycle touring intervention. *p* value derived from paired *t*-test. Data are shown as mean ±95% confident intervals.

Significant differences were obtained in CRF when comparing the baseline with the post-intervention (Δ = 0.455 ± 0.522; *p* = 0.016; Figure 3B), whereas no significant differences were noted in general fitness (Δ = 0.182 ± 0.603; *p* = 0.341; Figure 3A), muscular strength (Δ = 0.273 ± 0.467; *p* = 0.082; Figure 3C), speed/agility (Δ = 0.091 ± 0.539; *p* = 0.588; Figure 3D), and flexibility (Δ = 0.273 ± 0.467; *p* = 0.082; Figure 3E).

**Figure 4:**
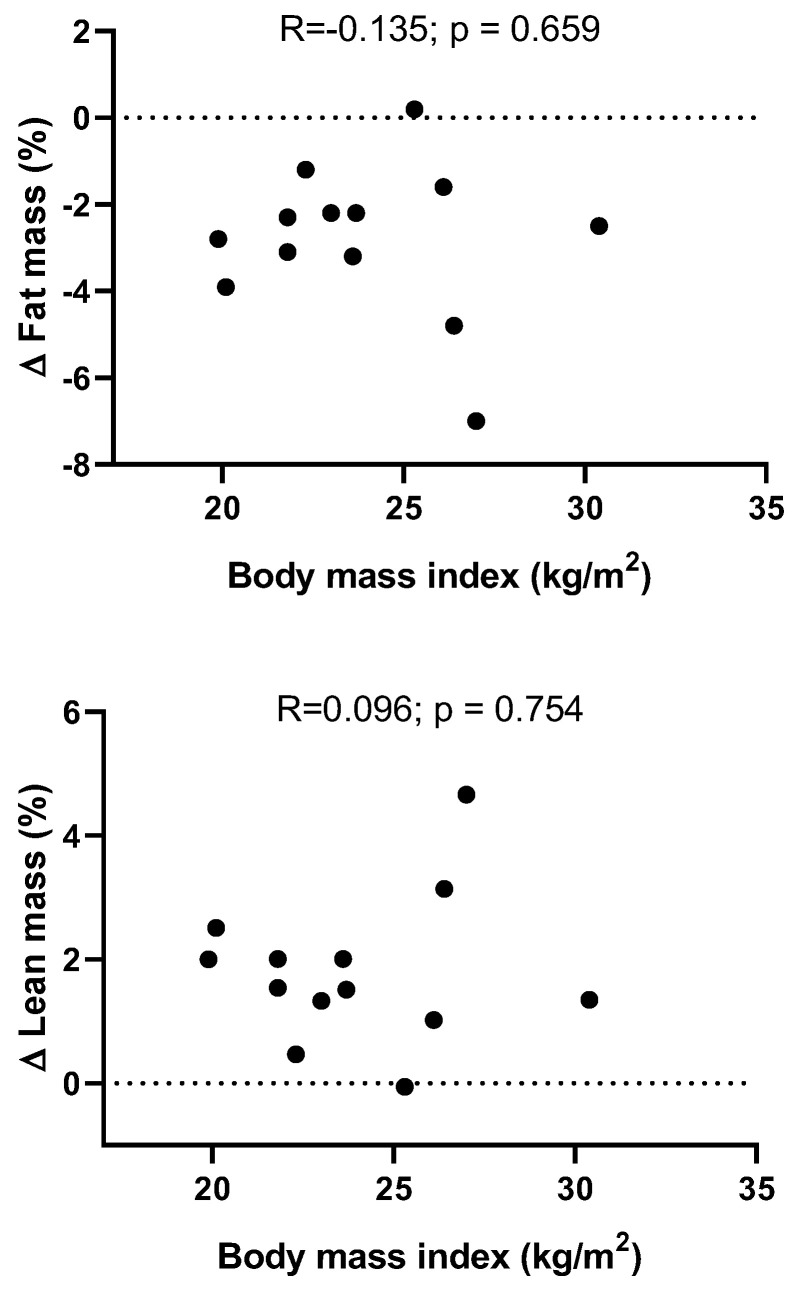
Associations between the difference in fat and lean mass between the baseline and post-intervention, and the body mass index at baseline.

We showed a lack of association between changes in fat mass (i.e., post-intervention minus baseline) and BMI baseline values (R = −0.135; *p* = 0.659; Figure 4). We also showed a lack of association between changes in lean mass (i.e., post-intervention minus baseline) and BMI baseline values (R = 0.096; *p* = 0.754; Figure 4).

Descriptive data regarding mean heart rate, perceived exertion, level of arousal, and pleasure by sessions can be found in Supplementary Figures S1 and S2.

## 4. Discussion

The main findings of this study were that a 7-day bicycle touring intervention induced a reduction of fat mass and an increase of lean mass in healthy individuals, while no changes were noted in either body weight or BMI. Moreover, a significant increase in CRF was observed after the intervention, whereas no significant differences were noted in general fitness, muscular strength, speed/agility, or flexibility.

Our results showed that a bicycle touring intervention did not change BMI, but that fat mass decreased and the lean mass increased in the short term. The current scientific literature has found a robust association of overweight and obesity with a higher risk of death or disease [39,40]. However, given that overweight and obesity assessment is based on BMI, a simultaneous increase of lean mass and decrease of fat mass could have a confusing interpretation, since it would not induce significant changes in BMI. The results of this research emphasize the importance of adding body composition measures apart from BMI. Indeed, previous studies have shown that a decrease in fat mass and an increase in lean mass could provide further improvements in human health [41,42].

In our study, we observed that a bicycle tour—whose average time was 6–7 h/day over 7 days, with a mean heart rate of 110–120 bpm—would decrease fat mass not only in the post-intervention, but also in re-test values. Thompson et al. [43] have previously reported that although PA could not lead to weight loss, it should not overlook the fact that regular exercise has numerous additional benefits in terms of body composition, which concurs with those results obtained in our study. These findings could be also explained by the lack of control of the participants’ diet, which could contribute to an isocaloric energy balance [44]. Our results are also in agreement with other studies that support the notion that PA can reduce fat mass and, consequently, obesity. Our results also concur with other cycling studies which evidence that commuter cycling decreases fat mass [27,45], and that even the use of e-bikes trends towards decreased fat mass too [46]. Finally, we observe that the difference between the baseline and re-test values compared with the difference between the baseline and post-intervention values was less remarkable in the former. This fact could be interpreted as the impact of the bicycle touring on body composition decreasing after few days.

We showed an increase in lean mass after the bicycle touring intervention, which disappeared when we compared the baseline and re-test values. This means that a 7-day bicycle tour could be considered as an effective strategy to increase lean mass in the short-term, but not in the long-term. Some previous studies have shown that higher lean mass is associated with a decrease in mortality risk, being significantly lower if it is associated with a low-fat mass status [42,47]. Likewise, it seems that the measurement of body composition (i.e., lean mass and fat mass) is necessary to understand whether an individual presents a healthy status, since there are specific situations in which weight loss is caused by a decrease of lean mass—increasing in this way all-cause mortality risk [41]. However, in this study, we observed an increase in lean mass only after the intervention, which disappeared after 7 days. An increase in lean mass in response to moderate intensity aerobic training is not expected—thus, confounding factors, including the measurement technology, hydration status, or the lack of accuracy of bioelectrical impedance for assessing body composition in athletes, could explain the present findings [48].

Even though body composition has been positioned as a very important marker of health status, much of the scientific literature concerning health-related physical fitness is focused on CRF as a predictor of mortality, morbidity, and disease risk factors [49,50,51,52,53,54]. In this study, we showed a significant improvement of CRF after the intervention. Farrell et al. [54] evidences that CRF remains significantly and inversely associated with mortality risk in men when comparing a contemporary cohort study with its impact on an earlier cohort. Some studies have suggested that when aiming to reduce mortality risk, we should focus on CRF level rather than BMI [51,52], while other studies evidence that both CRF and fatness are related to mortality and cardiovascular disease risk factors [48,50]. However, there is a wide consensus that a high fitness level is associated with a low risk of mortality and morbidity, regardless of weight or body composition [48,49,50,51,52]. Our study showed an increase in CRF and a decrease in fatness, which ensures in any case an improvement of the participants’ health. These results concur with those reported by previous studies performed with e-bikes [31,46] and in commuter cycling [26,27,45].

The present intervention is about bicycle touring, which has many distinctive characteristics, since it is an activity usually carried out at specific moments (e.g., holidays) that are not part of a daily routine [15]. Our intervention was conducted over an 8 day period, where the participants pedalled for 7 days for 6–7 h a day, which differs from others interventions in length terms (i.e., more hours a day, more days a week—but lower period duration compared with others [11,31,42,44,45,46]). Due to the lack of previous information and the promising results obtained from this research, future research lines on bicycle touring should focus on different aspects; diet should be included as a variable, as it would help us to understand the results on body composition, and the adherence to cycling after the intervention should be measured to evaluate the long-term benefits on health levels [55]. In addition, promoting cycling as a mode of transportation has strong practical implications in our society, since it has been recommended by the WHO during COVID-19—due to the ability to cycle while physically distancing [56]—and it has a high potential to reduce the impact of global warming [19] as a tool against climate change.

Some limitations of the present study need to be acknowledged. Firstly, we did not include a control group and, therefore, the improvements in body composition and physical fitness could not be definitively attributed to the bicycle touring intervention. Secondly, the body height measurement was self-reported once, and its value remained constant in the three measurement points, while the follow-up body composition measurement was performed prior to 24 h after the last exercise. Thirdly, there were daily facts not reported in the route (i.e., mechanical problems, personal needs, voluntary stops, etc.). Fourthly, physical fitness was subjectively assessed and, therefore, future studies should be implemented measuring this dimension with objective tests. Fourthly, nutrition during the intervention was not controlled and this fact could bias body composition results. Fifthly, wind and rain data during the stages were not registered. On the other hand, as the main strength of the present study, it is important to emphasize the novelty of a bicycle touring intervention carried out in an itinerant way, as a new research field that has not been previously studied.

## 5. Conclusions

In conclusion, a 7-day bicycle touring intervention induced a reduction in fat mass and an increase in lean mass in healthy individuals, while no changes were noted in body weight. Moreover, a significant increase in CRF was observed after the intervention, whereas no significant differences were noted in general fitness, muscular strength, speed/agility, or flexibility.

## Figures and Tables

**Table 1:** Descriptive parameters.

	All (*n* = 13)		Men (*n* = 10)		Women (*n* = 3)		*p*
Age (Years)	35.62	(11.68)	36.70	(12.49)	32.00	(9.54)	0.564
*Anthropometry and body composition*							
Body weight (kg)	75.10	(14.58)	80.35	(12.16)	57.60	(3.47)	**<0.001**
Body mass index (kg/m^2^)	23.95	(2.99)	24.36	(3.31)	22.60	(0.98)	0.395
Fat mass (%)	20.12	(6.35)	18.00	(4.91)	27.17	(6.07)	**0.020**
Lean mass (%)	45.09	(4.13)	46.67	(2.69)	39.83	(3.97)	**0.005**
*Physical fitness*							
General fitness	2.55	(0.69)	2.75	(0.46)	2.00	(1.00)	0.109
Cardiorespiratory fitness	2.27	(0.90)	2.50	(0.76)	1.67	(1.15)	0.186
Muscular strength	2.46	(0.52)	2.50	(0.53)	2.33	(0.58)	0.662
Speed/Agility	2.36	(0.81)	2.38	(0.52)	2.33	(1.53)	0.967
Flexibility	1.73	(0.90)	1.38	(0.52)	2.67	(1.15)	0.185

Statistically significant values (*p* < 0.05) are shown in bold. Differences between sexes were examined by unpaired sample *t*-test. Data are expressed as mean (standard deviation).

## Data Availability

Data are available upon reasonable request.

## Supplementary Materials

The following supporting information can be downloaded at: https://www.mdpi.com/article/10.3390/ijerph19052550/s1, Figure S1: Evolution of the mean heart rate and perceived exertion during the 7 days of the intervention (data presented individually per participant), Figure S2: Evolution of the level of arousal and pleasure during the 7 days of intervention measured with the Affective Slider questionnaire (data presented individually by participant).
