# Supplementary material for: Bicycle Touring 480 km in Seven Days: Effects on Body Composition and Physical Fitness—A Case Study
Source: Int J Environ Res Public Health. 2022 Feb 23;19(5):2550. doi: 10.3390/ijerph19052550 (PMC8909636; doi:10.3390/ijerph19052550)
Supplement: Supplementary file 1 [file ijerph-19-02550-s001.zip › ijerph-1525887-supplementary.pdf]

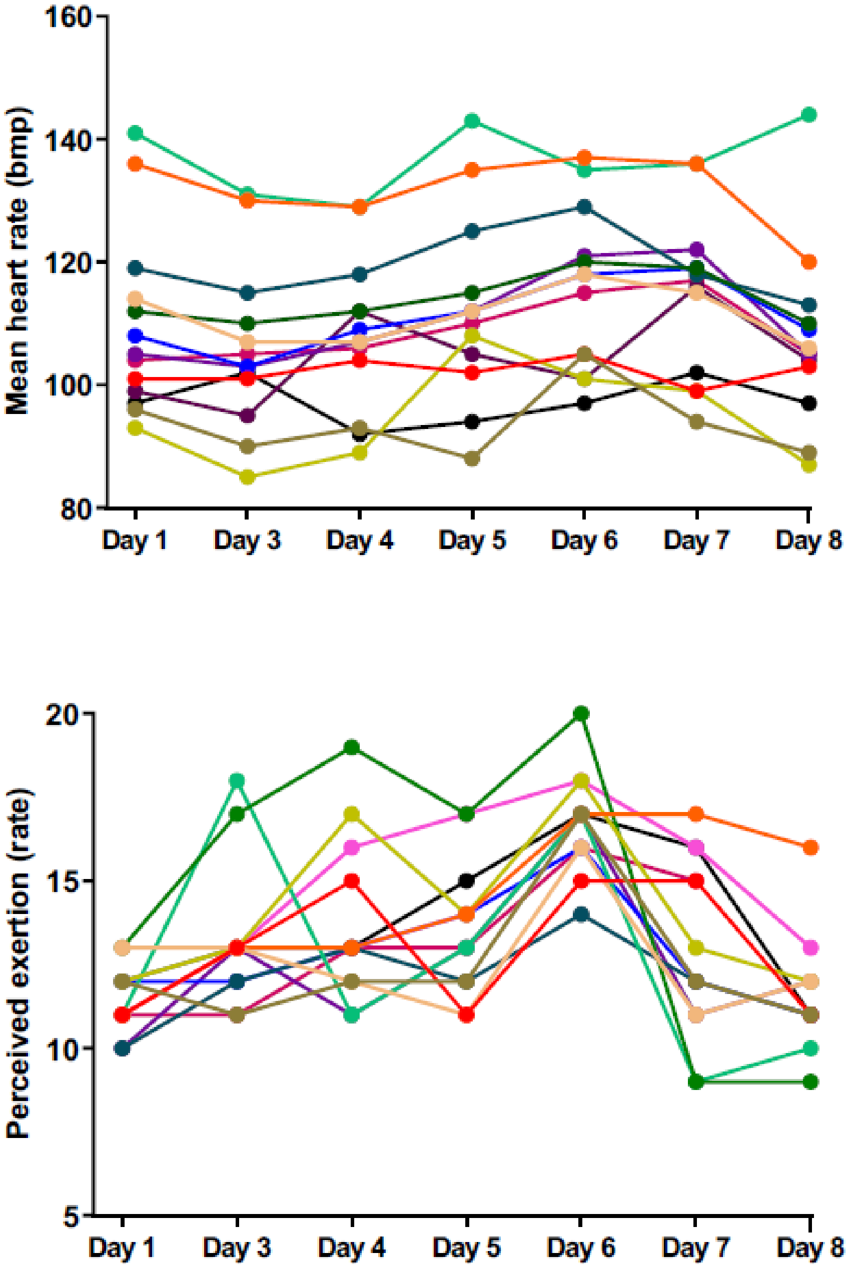

Figure S1. Evolution of the mean heart rate and perceived exertion during the 7 days of the intervention (data presented individually per participant)

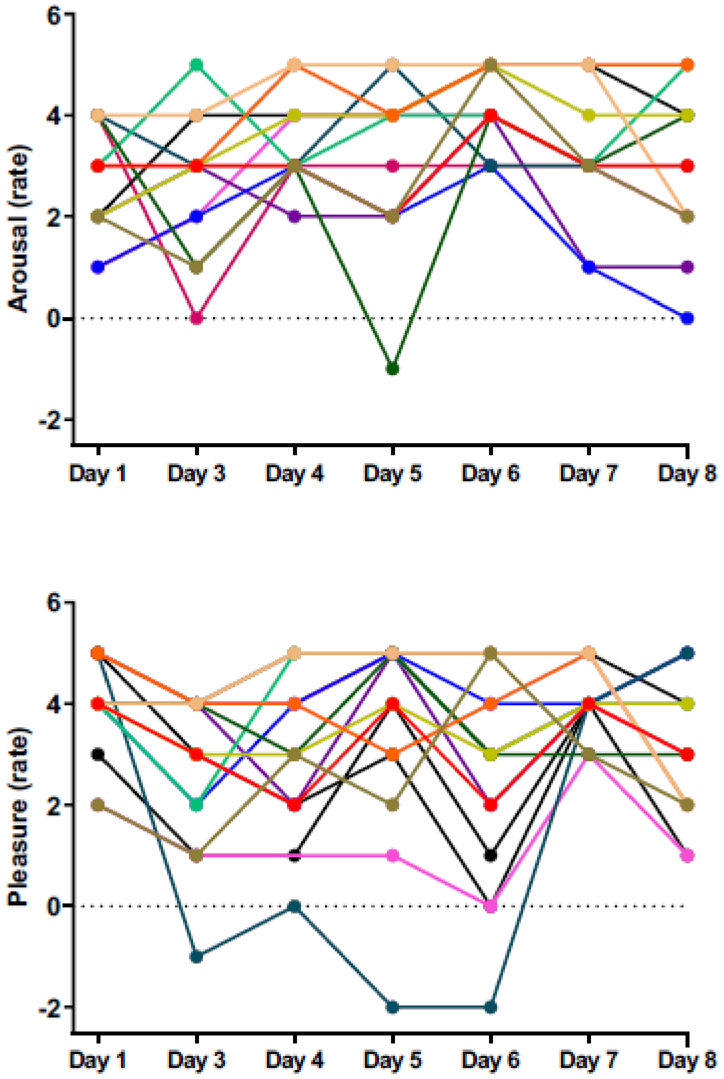

Figure S2. Evolution of the level of arousal and pleasure during the 7 days of intervention measured with the Affective Slider questionnaire (data presented individually by participant).
